# Reduction of in-hospital mortality after implementation of a pediatric trauma unit for children with moderate and severe trauma at Baca Ortiz Children's Hospital. Quito–Ecuador

**DOI:** 10.3389/fped.2026.1800573

**Published:** 2026-05-21

**Authors:** Frances Fuenmayor, Xavier Proaño, Rosa Quiguantar, Silvia Montaluisa, Fernanda Parra, Santiago Chavez, Marcelo Guerrero, Luis Fuenmayor-González

**Affiliations:** 1 Emergency Department, Baca Ortíz Pediatric Hospital, Quito, Ecuador; 2 School of Medicine, University of the Americas, Quito, Ecuador; 3 Intensive Care Medicine, Pontifical Catholic University of Ecuador, Quito, Ecuador; 4 School of Public Health, Washington University in St. Louis, St. Louis, MO, United States

**Keywords:** BIG score, in-hospital mortality, middle-income countries, pediatric trauma, quality improvement, trauma systems

## Abstract

Pediatric trauma is one of the leading causes of childhood morbidity and mortality worldwide. Optimizing early management in specialized units may substantially improve survival and functional outcomes. We therefore evaluated the impact of implementing a specialized Pediatric Trauma Unit on in-hospital mortality among children with moderate and severe trauma at Baca Ortiz Children's Hospital (HPBO) in Quito, Ecuador.

**Methods:**

We conducted a retrospective cohort study with historical controls including pediatric patients with moderate or severe trauma admitted before (*n* = 104) and after (*n* = 183) implementation of the Pediatric Trauma Unit. Descriptive statistics were used to summarize demographic and clinical characteristics. Chi-square and Fisher's exact tests were applied to compare categorical variables. Logistic regression was performed to assess the association between unit implementation and in-hospital mortality, adjusting for clinical severity scores and other potential confounders.

**Results:**

In-hospital mortality decreased from 16.3% in the pre-implementation period to 3.3% after implementation of the Pediatric Trauma Unit. Unit implementation was associated with an 88% reduction in the odds of death (adjusted OR 0.12; 95% CI 0.04–0.36; *p* < 0.001). Deceased patients had significantly higher pre-implementation BIG scores than survivors (*p* = 0.02). Survival among patients with abdominal trauma also improved after protocol implementation (*p* = 0.01).

**Conclusions:**

Implementation of a specialized Pediatric Trauma Unit at Baca Ortiz Children's Hospital was associated with a marked reduction in in-hospital mortality among children with moderate and severe trauma. These findings support the development and strengthening of pediatric trauma care systems in similar settings.

## 1 Introduction

Pediatric trauma is a major global health problem because of the substantial morbidity and mortality it causes worldwide (1). The World Health Organization and UNICEF estimated that about 950,000 children and adolescents younger than 18 years die each year from injury-related causes, and that almost 90% of these deaths are due to unintentional injuries (1). Across most mechanisms, males are disproportionately affected (1).

In Ecuador, updated national pediatric trauma registries are scarce (2). Available Ecuadorian literature suggests a substantial pediatric trauma burden and a predominance of traffic-related injuries among minors (2). At Baca Ortiz Children's Hospital in Quito, a series of severe pediatric traumatic brain injuries found that 45.8% were caused by traffic accidents and 43.8% by falls; survival among children transported by ambulance reached 79.5% (3). In Cuenca, another pediatric traumatic brain injury series also showed male predominance and identified falls and traffic accidents as the most frequent mechanisms (4). These Ecuadorian data were generated in a context in which dedicated pediatric trauma infrastructure was still being proposed locally (5). Standardizing trauma care across South and Central America remains difficult because of limited training, shortages of resources and equipment, weak implementation of standardized protocols, deficient documentation, transport and logistics barriers, and unequal access to organized trauma systems (6, 7). In response, Ecuador has developed technical and operational documents to prioritize rapid initial management and referral of critically ill or injured patients. Among these, the Código Rojo activation instructions establish rapid identification and transfer to higher-complexity hospitals according to clinical need and institutional capability (8, 9). In pediatric trauma, activation criteria include hemodynamic instability, sudden deterioration in Glasgow Coma Scale score, severe traumatic brain injury, penetrating thoracoabdominal trauma, and moderate-to-severe traumatic brain injury requiring urgent surgery (8, 9). Within this framework, Baca Ortiz Children's Hospital (HPBO, for its initials in Spanish), a national pediatric referral hospital in Quito, inaugurated in May 2023 the country's first specialized Pediatric Trauma and Shock Unit in the emergency department (10). According to official and institutional documents, the unit includes a dedicated trauma/shock area with eight beds, trained personnel, specialized protocols, and ventilatory, hemodynamic, and neuromonitoring support, with the goal of reducing morbidity and mortality (10, 11). This unit therefore represents one of the first structured models of specialized pediatric trauma care publicly documented in Ecuador. The objective of this study was to evaluate the impact of implementing this Pediatric Trauma Unit on in-hospital mortality among children with moderate and severe trauma at HPBO. The Baca Ortiz Children's Hospital is located in Quito, the capital of Ecuador, and has a total capacity of 356 licensed beds. It has an Emergency Department that currently receives more than 1,300 trauma patients per year. This center houses the first pediatric trauma unit at the national level, which was created in May 2023. It was designed to care for 8 critically ill patients diagnosed with trauma and severe polytrauma. Each bed is equipped with state-of-the-art technology, latest-generation mechanical ventilation, hemodynamic monitoring, invasive and noninvasive neuromonitoring, as well as updated and standardized protocols based on the latest available evidence (11). For the treatment of these patients, there is a team of specialist physicians in Pediatric Emergency Medicine, pediatric intensivists, neurosurgery, traumatology, and neurology, in addition to a fully equipped operating room, laboratory, and pharmacy available 24 h a day, 365 days a year (11). The flow of patients treated in the trauma unit since its creation has been 458 (10%) in 2023, 744 (11%) in 2024, and 790 (7.4%) in 2025 (12).

## 2 Methods

### 2.1 Study design and setting

We conducted an analytical retrospective cohort study with historical controls at HPBO. The study compared outcomes of pediatric trauma patients admitted before and after implementation of a specialized Pediatric Trauma Unit.

### 2.2 Study periods and population

Two time periods were analyzed: the pre-implementation period, from August 2021 to February 2022, when patients with trauma were managed in the non-specialized Emergency Unit; and the post-implementation period, from February 2022 to August 2023, after implementation of the Pediatric Trauma Unit. The study population consisted of 287 pediatric patients with trauma: 104 admitted during the pre-implementation period and 183 during the post-implementation period. Pediatric Trauma Unit and intervention.

The Pediatric Trauma Unit was implemented to centralize and standardize the care of children with moderate and severe trauma. During the pre-implementation period, pediatric trauma care was provided within a conventional pediatric emergency department. Under this model, care was decentralized, and the initial evaluation was performed by general on-duty staff without the continuous presence of a specialized trauma team. There were no standardized activation criteria based on pediatric-specific physiological parameters; instead, the involvement of specialists, such as pediatric surgeons or intensivists, was managed through individual consultations at the discretion of the attending physician. The workflow for diagnostic imaging and laboratory tests followed standard emergency department waiting times, as there was no defined priority pathway for moderate or severe trauma. Response times for specialist evaluation and access to critical interventions were not protocolized and depended on institutional availability at the time of admission.

In the post-implementation period, patients who met national Código Rojo criteria for major trauma were either triaged directly to the unit or transferred from the Emergency Department after initial stabilization (8, 9). The unit comprises a dedicated resuscitation area and a multidisciplinary team including pediatric surgeons, emergency physicians, intensivists, anesthesiologists, trauma nurses, and respiratory therapists, with 24/7 availability (10, 11). Key features of the intervention included: (1) standardized activation criteria based on the Pediatric and Adult Red Code Manual; (2) predefined resuscitation and imaging algorithms; (3) early, systematic involvement of pediatric surgery and intensive care in the initial assessment; and (4) implementation of structured monitoring and escalation protocols for high-risk injuries such as abdominal trauma and severe TBI. No major technological investments were introduced concurrently; the intervention primarily involved organizational restructuring and protocol-driven care.

### 2.3 Eligibility criteria

Inclusion criteria were age between 0 months and 15 years 11 months 29 days; diagnosis of moderate or severe trauma, including polytrauma, according to the criteria established in the national Código Rojo Manual (8, 9); and admission to either the non-specialized Emergency Unit during the pre-implementation period or the Pediatric Trauma Unit during the post-implementation period at HPBO. Exclusion criteria were incomplete medical records and readmissions for the same trauma episode, in which case only the first admission was considered. Variables and definitions.

The primary outcome was in-hospital mortality during the index admission. The main exposure variable was admission during the post-implementation period, defined as care in the Pediatric Trauma Unit, versus admission during the pre-implementation period, defined as care in the non-specialized Emergency Unit.

Covariates included age and sex; geographic region of origin, classified according to the patient's place of residence as Coast, Highlands, or Amazon; presence of comorbidities, defined as pre-existing chronic medical conditions documented in the medical record; and admission diagnosis, categorized as abdominal trauma, polytrauma, moderate traumatic brain injury (TBI), severe TBI, thoracic trauma, or other diagnoses. Each patient was assigned to a single diagnostic category according to the main injury identified at admission.Clinical severity was assessed using three scores. The Trauma Index was routinely calculated at admission for trauma patients at HPBO and reflected anatomic and physiological injury severity. The BIG score was calculated as base deficit + [2.5 × international normalized ratio (INR)] + (15 - Glasgow Coma Scale), as originally described by Borgman et al. (13). Pediatric Index of Mortality 3 (PIM3) was computed from variables collected within the first hour of pediatric intensive care unit admission, following the original model specification described by Straney et al. (14). Statistical analysisContinuous variables were summarized as medians and 25th and 75th percentiles or means and standard deviations, as appropriate. Categorical variables were expressed as frequencies and percentages. The Shapiro–Wilk test was used to assess normality of continuous variables. Group comparisons between pre- and post-implementation periods were performed using the Mann–Whitney U test for continuous variables and the chi-square or Fisher's exact test for categorical variables, as appropriate.

Multivariable logistic regression was employed to estimate the association between the implementation of the specialized pediatric trauma unit and hospital mortality. Variables with *p* < 0.20 in univariate analyses and those considered clinically relevant, such as age, sex, and injury severity scores, were considered for inclusion in the model. Model assumptions were rigorously tested; multicollinearity among independent variables was assessed using Variance Inflation Factors (VIF), with values < 10 considered acceptable. Model discrimination was evaluated using the area under the receiver operating characteristic curve (C-statistic), where a value > 0.80 indicated excellent discriminative power. Calibration was assessed using the Hosmer–Lemeshow goodness-of-fit test, with a *p*-value > 0.05 indicating adequate agreement between observed and predicted outcomes. Finally, model diagnostics were performed by inspecting deviance residuals plotted against predicted probabilities to identify potential influential observations and ensure overall model fit. Results are reported as odds ratios with 95% confidence intervals. A two-sided *p* value < 0.05 was considered statistically significant. Statistical analysis was performed using SPSS version 26.0 and STATA 19.

### 2.4 Ethical considerations

All data were anonymized in accordance with the Personal Data Protection Law of the Republic of Ecuador. The study protocol was reviewed and approved by HPBO's CEISH (Committee of Ethics and Research in Human Beings by its initials in Spanish) with the code No. 067-CEISH-HPBO-2024.

## 3 Results

As shown in Table 1, we did not find clearly statistically significant differences between deceased and surviving patients in terms of age, sex, presence of comorbidities, or use of invasive mechanical ventilation in either the pre- or post-implementation periods. A borderline difference was observed for the Amazon region in the pre-implementation period, where a higher proportion of deceased patients originated from this region compared with survivors (29.4% vs. 11.5%; *p* = 0.05). This difference was no longer apparent after implementation of the Pediatric Trauma Unit. Although these patterns may suggest differential vulnerability related to geographic origin, they should be interpreted with caution given the small number of deaths. Overall, baseline demographic and clinical variables did not show strong statistical evidence of imbalance between survivors and non-survivors within each study period.

**Table 1:** Clinical and demographic characteristics of participants by vital status and study period.

Variables	Pre-implementation (*n* = 104)		*p* value	Post-implementation (*n* = 183)		*p* value
	Death (*n* = 17)	Survival (*n* = 87)		Death (*n* = 6)	Survival (*n* = 177)	
Age (median, 25th and 75th percentile)	2 (2–3)	3 (2–3)	0.86^b^	2.5 (2–3)	3 (2–3)	0.64^b^
Males (n, %)	15 (88.2)	71 (81.6)	0.50	6 (100)	151 (85.3)	0.31
Comorbidities (n, %)	0 (0)	3 (3.4)	0.43^a^	0 (0)	2 (1.1)	0.79^a^
Invasive Mechanical Ventilation Usage (n, %)	14 (82.4)	57 (65.5)	0.17	6 (100)	131 (74)	0.14
Geographical regions of participants						
Coast (n, %)	3 (17.6)	12 (13.8)	0.70^a^	1 (16.7)	22 (12.4)	0.55^a^
Highlands (n, %)	9 (52.9)	65 (74.7)	0.07	4 (66.7)	139 (78.5)	0.61^a^
Amazon (n, %)	5 (29.4)	10 (11.5)	0.05	1 (16.7)	16 (9)	0.44^a^

a Fisher's exact test.

b Median (percentiles); *p* value from Mann–Whitney U test.

As shown in Table 2, severity scores were generally higher in non-survivors than in survivors in both study periods. In the pre-implementation period, non-survivors had significantly higher BIG scores compared with survivors [median 21.0 [10–30] vs. 12.9 [5–20.1]; *p* = 0.02]. A similar pattern was observed after implementation [median 21.5 [15.8–25] vs. 17.0 [8–22.6]], although this difference did not reach statistical significance (*p* = 0.28), likely due to the small number of deaths. In contrast, differences in the Trauma Index and PIM3 between survivors and non-survivors were small and not statistically significant in either period.

**Table 2:** Performance of severity scores by vital status and study period.

Clinical Scores	Pre-implementation (*n* = 104)		*p* value	Post-implementation (*n* = 183)		*p* value
	Death (*n* = 17)	Survival (*n* = 87)		Death (*n* = 6)	Survival (*n* = 177)	
Trauma Index, median (25th and 75th percentiles)	6 (4–6)	6 (3–8)	0.80^a^	5.5 (5–6.7)	6 (4–8)	0.75^a^
PIM3, median (25th and 75th percentiles)	6 (2–26)	5 (2.1–22.2)	0.61^a^	4.8 (2.1–9.8)	5 (2–17)	0.55^a^
BIG score, median (25th and 75th percentiles)	21 (10–30)	12.9 (5–20.1)	0.02^a^	21.5 (15.8–25)	17 (8–22.6)	0.28^a^

a Median (25th and 75th percentiles); *p* value from Mann–Whitney U test.

Regarding admission diagnoses (Table 3), abdominal trauma was the only category that showed a statistically significant association with mortality in both study periods. In the pre-implementation period, 17.6% of deaths vs. 1.1% of survivors had abdominal trauma (*p* = 0.01), while in the post-implementation period the corresponding proportions were 33.3% and 2.8% (*p* = 0.01). These findings suggest that abdominal trauma may represent a particularly high-risk subgroup in this cohort, although the small number of cases warrants cautious interpretation.

**Table 3:** Admission diagnoses of participants by vital status and study period.

Diagnosis	Pre-implementation (*n* = 104)		*p* value	Post-implementation (*n* = 183)		*p* value
	Death (*n* = 17)	Survival (*n* = 87)		Death (*n* = 6)	Survival (*n* = 177)	
Abdominal trauma. *n* (%)	3 (17.6)	1 (1.1)	0.01^a^	2 (33.3)	5 (2.8)	0.01^a^
Polytrauma. *n* (%)	1 (5.9)	11 (12.6)	0.68^a^	0 (0)	20 (11.3)	0.38^a^
Moderate Traumatic Brain Injury *n* (%)	1 (5.9)	17 (19.5)	0.29^a^	0 (0)	31 (17.5)	0.59^a^
Severe Traumatic Brain Injury *n* (%)	12 (70.6)	55 (63.2)	0.56	4 (66.6)	115 (65)	0.93^a^
Thoracic Trauma *n* (%)	0 (0)	1 (1.1)	0.65^a^	0 (0)	2 (1.1)	0.79^a^
Others *n* (%)	0 (0)	2 (2.3)	0.52^a^	0 (0)	4 (2.3)	0.71^a^

a Fisher's exact test.

Severe traumatic brain injury (TBI) was the most frequent diagnosis among non-survivors in both periods; however, it did not differ significantly between survivors and non-survivors, likely because it was also very common among survivors, limiting its discriminatory power in this analysis. For polytrauma and moderate TBI, no clear patterns or statistically significant differences were observed. Thoracic trauma and the remaining diagnostic categories included very few cases, precluding firm conclusions about their association with mortality.

In the multivariable logistic regression model (Table 4), implementation of the Pediatric Trauma Unit was strongly associated with lower in-hospital mortality. After adjustment for age, sex, admission diagnosis, geographic region, and severity scores, admission in the post-implementation period was associated with an 88% reduction in the odds of death (adjusted OR 0.12; 95% CI 0.04–0.36; *p* < 0.001).

**Table 4:** Multivariable logistic regression for in-hospital mortality.

Variable	β	SE	Z	*p* value	aOR	95% CI
Trauma Unit Implementation	−2.11	0.55	−3.79	< 0.001	0.12	0.04–0.36
Age (ref: 1 year)						
2 years old	1.17	1.44	0.81	0.41	3.24	0.19–55.28
3 years old	0.48	1.41	0.34	0.73	1.62	0.10–26.32
Female sex (ref: male)	−1.57	0.91	−1.72	0.08	0.20	0.03–1.24
Comorbidities^a^	–	–	–	–	–	–
Polytrauma (ref: other diagnoses)	−3.19	1.28	−2.49	0.013	0.04	0.003–0.50
Moderate TBI (ref: other diagnoses)	−3.36	1.22	−2.74	0.006	0.03	0.003–0.38
Severe TBI (ref: other diagnoses)	−2.31	0.81	−2.86	0.004	0.09	0.02–0.48
Region (ref: Coast)						
Highlands	0.70	0.76	0.09	0.61	1.07	0.23–4.85
Amazon	1.39	0.93	1.49	0.11	4.02	0.64–25.06
Trauma Index	0.02	0.07	0.38	0.70	1.02	0.88–1.19
PIM 3	−0.01	0.01	−0.85	0.39	0.98	0.95–1.01
BIG Score	0.05	0.01	2.83	0.005	1.05	1.01–1.10

a The variable “Comorbidities” was excluded from the multivariable model due to a lack of variability (zero deaths in the comorbid group).

The final logistic regression model demonstrated excellent performance and stability. All independent variables showed no evidence of multicollinearity, with VIF values well below the established threshold of 10. Model discrimination was high, yielding a C-statistic of 0.8556. Calibration was adequate, as indicated by a non-significant Hosmer-Lemeshow test (*χ*^2^ = 10.35; *p* = 0.2416), suggesting no evidence of lack of fit. The model's overall explanatory power was further supported by a McFadden's pseudo-R-squared of 0.26. Diagnostic plots of deviance residuals against predicted probabilities confirmed a satisfactory fit, with no significant outliers or influential observations that substantially altered the model's estimates.

In absolute terms, overall mortality decreased from 16.3% (17/104) in the pre-implementation period to 3.3% (6/183) after implementation, representing a 13-percentage-point reduction and an approximate 80% relative reduction in risk.

Among the covariates, the BIG score remained independently associated with mortality in the adjusted model: each additional point in the BIG score increased the odds of in-hospital death by 5% (OR 1.05; 95% CI 1.01–1.10; *p* = 0.005). In contrast, the Trauma Index and PIM3 did not show statistically significant associations with mortality in this cohort.

In the regression model, polytrauma and moderate or severe traumatic brain injury were associated with very low odds ratios relative to the reference diagnosis. These estimates should not be interpreted as indicating a true protective effect. More likely, they reflect the choice of reference category and the small number of deaths within some diagnostic subgroups. Likewise, the estimate for comorbidities was unstable and not clinically interpretable. Overall, these results indicate that the model had limited precision for comparing individual diagnostic categories, so, these coefficients should be interpreted cautiously.

## 4 Discussion

In this retrospective cohort study with historical controls, implementation of a specialized Pediatric Trauma Unit at a tertiary referral hospital in Ecuador was associated with a marked reduction in in-hospital mortality among children with moderate and severe trauma. Taken together, these findings indicate that the effect of the Pediatric Trauma Unit should be interpreted as the impact of a bundled organizational intervention. The marked reduction in overall mortality after implementation, together with the persistence of this association in the adjusted model, is consistent with a beneficial effect of standardized activation, algorithm-based resuscitation and imaging, early multidisciplinary assessment, and structured monitoring of high-risk injuries. The disappearance of the pre-implementation mortality pattern among children from the Amazon region further suggests improved triage and referral coordination after implementation. In addition, the independent association between BIG score and mortality supports the value of systematic early physiological assessment within this model, while the persistent risk observed in abdominal trauma and the high frequency of severe TBI among non-survivors identify the subgroups in whom monitoring and escalation protocols are likely to be most relevant. Because the intervention was implemented as an integrated care package, the present analyses do not allow the independent contribution of each component to be quantified.

An important implication of our findings is that the mortality reduction observed after implementation should be interpreted considering the specific intervention components described in the Methods. Because no major technological investments were introduced concurrently, the observed benefit is more plausibly attributable to organizational restructuring and protocol-driven care. In this sense, the 88% adjusted reduction in the odds of death supports the value of a structured trauma model based on standardized activation, predefined resuscitation and imaging pathways, early involvement of pediatric surgery and intensive care, and formal monitoring and escalation procedures for high-risk injuries.

First, standardized activation criteria based on the Red Code Manual may have improved consistency in triage and timeliness of referral to specialized care. This interpretation is indirectly supported by the disappearance, after implementation of the pre-existing pattern of higher mortality among children from the Amazon region, although the small number of deaths requires caution. Second, the finding that the BIG score was the only severity measure independently associated with mortality is compatible with the usefulness of predefined resuscitation pathways and systematic early physiological assessment during the initial evaluation. Third, the relevance of early, systematic involvement of pediatric surgery and intensive care is supported by the overall survival benefit observed after introduction of a 24/7 multidisciplinary trauma unit, particularly in conditions such as abdominal trauma and severe TBI where prompt specialist assessment is clinically important. Fourth, abdominal trauma was the only diagnostic category significantly associated with mortality in both periods, and severe TBI remained the most frequent diagnosis among non-survivors, which reinforces the rationale for structured monitoring and escalation protocols in these high-risk subgroups. However, the present study did not collect process measures such as activation time, time to imaging, time to specialist assessment, or adherence to escalation algorithms, these findings should be interpreted as support for the intervention bundle rather than proof of the independent effect of each component.

Overall mortality decreased from 16.3% in the pre-implementation period to 3.3% after implementation, corresponding to an absolute reduction of 13 percentage points and an approximate 80% relative reduction in risk. In the multivariable logistic regression, admission in the post-implementation period remained independently associated with an 88% reduction in the odds of death after adjustment for demographic characteristics, admission diagnosis, geographic region, and severity scores. Among the covariates, the BIG score was the only severity index that showed an independent association with mortality.

Pediatric trauma poses specific challenges due to the unique anatomical and physiological characteristics of children, including smaller and more vulnerable airways, different patterns of blood volume distribution, and higher susceptibility to permanent neurological damage when critical injuries are not rapidly recognized and treated (15). Traumatic brain injury remains a leading cause of trauma-related morbidity and mortality in children and continues to require standardized, evidence-based management (15). In our cohort, demographic variables such as age, sex, and presence of comorbidities did not differ significantly between survivors and non-survivors in either period, nor were they independent predictors of mortality in the multivariable model. This suggests that, in this population, outcomes were more strongly driven by injury severity and timeliness of care than by baseline demographic characteristics. We also observed that an initial pattern of higher mortality among patients from the Amazon region was no longer evident after implementation, which may indicate improved coordination and more timely access to specialized care for children referred from remote areas. This pattern is also compatible with the possibility that standardized activation criteria and more structured referral pathways improved recognition and prioritization of severely injured children arriving from geographically remote areas. However, given the small number of deaths in these subgroups, this finding should be interpreted with caution and considered hypothesis-generating. Severity scoring systems are essential tools for early risk stratification in pediatric trauma. In our study, three scores were analyzed: the Pediatric Trauma Index, PIM3, and the BIG score. While the Trauma Index and PIM3 did not show significant differences between survivors and non-survivors, the BIG score behaved as expected. Before implementation, non-survivors had significantly higher BIG scores than survivors, and in the multivariable model, each additional point in the BIG score increased the odds of in-hospital death by 5%. This pattern is consistent with the original description of the BIG score and with later validation studies showing that the BIG score discriminates between survivors and non-survivors in pediatric trauma (13, 16). Our results support the use of the BIG score as a rapid and clinically useful risk-stratification tool in pediatric trauma settings, particularly during initial resuscitation. Abdominal trauma emerged as an especially vulnerable subgroup in our cohort. Before implementation of the Pediatric Trauma Unit, 17.6% of deceased patients vs. 1.1% of survivors had abdominal trauma; after implementation, the corresponding figures were 33.3% and 2.8% (both *p* = 0.01). Although the absolute number of cases was small, these patterns suggest that children with abdominal trauma may benefit substantially from structured protocols and specialized teams. This observation is consistent with reports that standardized pediatric management protocols for blunt abdominal or renal trauma can reduce resource use without increasing complication rates (17). This interpretation is especially relevant because abdominal trauma was explicitly targeted in the intervention design as a high-risk condition requiring structured monitoring and escalation, making this subgroup one of the clearest clinical correlations of the methodology described in our study. Together, these data highlight the importance of clearly defined pathways for the evaluation and management of abdominal injuries in pediatric trauma centers. The overall magnitude of mortality reduction observed in our study is broadly consistent with, although larger than, estimates reported in international literature. In a meta-analysis including children younger than 19 years, Moore et al. found that care in pediatric trauma centers was associated with a 41% reduction in mortality compared with adult or mixed trauma centers (pooled OR 0.59; 95% CI 0.46–0.76) (18). Our adjusted OR of 0.12 for mortality after implementation, likely reflects several factors, including the use of historical controls, differences in baseline resource availability, and the magnitude of organizational change associated with creating a specialized Pediatric Trauma Unit in a setting that previously lacked such a structure. Importantly, because no major technological investments were introduced concurrently, the observed improvement is more consistent with the clinical value of reorganization, protocol standardization, and earlier multidisciplinary decision-making than with equipment-related changes. Rather than indicating that our model surpasses those in high-income countries, these findings underscore the potential impact of implementing organized pediatric trauma care in resource-constrained environments where baseline mortality is higher. The multivariable model also provides important insights and requires careful interpretation. Beyond the effect of unit implementation and the BIG score, other covariates did not show statistically significant associations with mortality. The very low odds ratios obtained for polytrauma and moderate or severe TBI, which could be interpreted as protective if taken at face value, almost certainly reflect the choice of reference category and the limited number of deaths in specific diagnostic groups rather than a true protective effect of these conditions. Likewise, the comorbidity estimate was unstable and could not be interpreted reliably. From a clinical perspective, these results mean that the model is informative for the overall effect of unit implementation, but not for drawing firm conclusions about the independent effect of each diagnostic subgroup. Equity considerations are another relevant aspect of our findings. Before implementation, children from remote regions such as the Amazon appeared to face a higher risk of mortality, whereas after implementation this gap was no longer evident. Although our data is insufficient to draw definitive conclusions, they suggest that strengthening referral pathways and centralized pediatric trauma care may contribute to reducing territorial inequities in access to timely, high-quality treatment. This aligns with broader regional efforts to organize trauma systems and referral networks in Latin America (7). This study has several strengths. It focuses exclusively on the pediatric population in a middle-income country, uses a historical control group that allows the effect of a major organizational intervention to be assessed, and applies multivariable analysis to adjust for several key confounders. The results are consistent with the direction of effects reported in observational studies and meta-analyses examining pediatric trauma centers in other regions (18), which supports the external validity of our findings for similar contexts. However, important limitations must be acknowledged. First, the retrospective design relies on the completeness and accuracy of medical records and precludes causal inferences. Second, the relatively small number of deaths, particularly in the post-implementation period, limits statistical power and contributes to unstable estimates for some variables in the regression model. In addition, we did not collect process indicators such as Código Rojo activation time, time to imaging, time to pediatric surgery or PICU assessment, or adherence to monitoring and escalation protocols; therefore, we could not estimate the independent contribution of each intervention component, and our findings should be interpreted as the effect of the Pediatric Trauma Unit as a bundled organizational intervention. Third, residual confounding by unmeasured factors, such as changes in staffing, informal training, parallel institutional initiatives, cannot be ruled out. Fourth, we focused on in-hospital mortality and did not assess long-term functional outcomes or quality of life, which are crucial when evaluating the full impact of trauma care on children. Despite these limitations, the magnitude and consistency of the observed mortality reduction strongly suggest that implementation of a specialized Pediatric Trauma Unit contributed to improved survival among children with moderate and severe trauma at our institution. This experience may serve as a reference for other hospitals in Ecuador and the wider region seeking to organize pediatric trauma care. Future research should aim to include larger multicenter cohorts, incorporate standardized measures of long-term functional outcomes and quality of life, and explore the cost-effectiveness and equity impact of pediatric trauma networks in low- and middle-income settings.

## 5 Conclusions

Our results also suggest that the observed benefit was consistent with a protocol-driven model centered of standardized activation criteria, predefined resuscitation and imaging algorithms, early involvement of pediatric surgery and intensive care, and structured monitoring and escalation for high-risk injuries such as abdominal trauma and severe TBI. Although the present study does not allow the independent effect of each component to be isolated, it provides important evidence that organizational restructuring, even in the absence of major technological investments, can improve survival in this highly vulnerable population.

Implementation of a specialized Pediatric Trauma Unit at Baca Ortiz Children's Hospital was associated with a significant reduction in in-hospital mortality among children with moderate and severe trauma. These findings support the development and implementation of similar pediatric trauma care models at national and regional levels.

Our results also suggest that certain subgroups such as children with abdominal trauma may derive particular benefit from structured, protocol-driven management.

In summary, this study provides important evidence that organizing trauma care within a dedicated pediatric unit can significantly improve survival in a highly vulnerable population. The use of prognostic tools such as the BIG score, the adoption of clear, standardized protocols and the availability of a multidisciplinary team around the clock appear to be key components for optimizing clinical outcomes. The experience of Baca Ortiz Children's Hospital illustrates how organizational restructuring, even in the absence of major technological investments, can substantially improve health outcomes for injured children.

## Data Availability

The original contributions presented in the study are included in the article/Supplementary Material, further inquiries can be directed to the corresponding author.
